# The Impacts of Computer-Aided Detection of Colorectal Polyps on Subsequent Colonoscopy Surveillance Intervals: Simulation Study

**DOI:** 10.2196/42665

**Published:** 2023-02-10

**Authors:** Ka Luen Thomas Lui, Sze Hang Kevin Liu, Kathy Leung, Joseph T Wu, Ann G Zauber, Wai Keung Leung

**Affiliations:** 1 Department of Medicine, School of Clinical Medicine Li Ka Shing Faculty of Medicine The University of Hong Kong Hong Kong Hong Kong; 2 WHO Collaborating Centre for Infectious Disease Epidemiology and Control, School of Public Health Li Ka Shing Faculty of Medicine The University of Hong Kong Hong Kong Hong Kong; 3 Department of Epidemiology and Biostatistics Memorial Sloan Kettering Cancer Center New York, NY United States

**Keywords:** artificial intelligence, surveillance colonoscopy, colonic polyp, polyp, colonoscopy, computer-aided, detect, adenoma, endoscopic, endoscopy, simulation, simulated, surveillance

## Abstract

**Background:**

Computer-aided detection (CADe) of colorectal polyps has been shown to increase adenoma detection rates, which would potentially shorten subsequent surveillance intervals.

**Objective:**

The purpose of this study is to simulate the potential changes in subsequent colonoscopy surveillance intervals after the application of CADe in a large cohort of patients.

**Methods:**

We simulated the projected increase in polyp and adenoma detection by universal CADe application in our patients who had undergone colonoscopy with complete endoscopic and histological findings between 2016 and 2020. The simulation was based on bootstrapping the published performance of CADe. The corresponding changes in surveillance intervals for each patient, as recommended by the US Multi-Society Task Force on Colorectal Cancer (USMSTF) or the European Society of Gastrointestinal Endoscopy (ESGE), were determined after the CADe was determined.

**Results:**

A total of 3735 patients who had undergone colonoscopy were included. Based on the simulated CADe effect, the application of CADe would result in 19.1% (n=714) and 1.9% (n=71) of patients having shorter surveillance intervals, according to the USMSTF and ESGE guidelines, respectively. In particular, all (or 2.7% (n=101) of the total) patients who were originally scheduled to have 3-5 years of surveillance would have their surveillance intervals shortened to 3 years, following the USMSTF guidelines. The changes in this group of patients were largely attributed to an increase in the number of adenomas (n=75, 74%) rather than serrated lesions being detected.

**Conclusions:**

Widespread adoption of CADe would inevitably increase the demand for surveillance colonoscopies with the shortening of original surveillance intervals, particularly following the current USMSTF guideline.

## Introduction

The application of computer-aided detection (CADe) of colorectal polyps has been consistently shown to increase the adenoma detection rate as well as the total number of adenomas detected per patient during a real-time colonoscopy [1-46]. A recent modeling study further suggested that the use of CADe could potentially reduce colorectal cancer mortality [47]. However, CADe tends to detect more small lesions, which could lead to possible overdiagnosis as the future cancer risk of these small lesions remains debatable [48-51]. However, with the increase in the number of colonic lesions detected by CADe, the subsequent surveillance colonoscopy intervals may have to be shortened for some patients, as the current guideline was also based on the number of lesions detected [52].

This study aims to determine the potential impact on the subsequent surveillance intervals of the routine use of CADe for colorectal polyp detection. Based on the baseline colorectal polyp and adenoma distribution of our patients, we simulated the projected increase in all colonic lesions detected with the application of CADe and the corresponding changes in surveillance intervals, according to the US Multi-Society Task Force on Colorectal Cancer (USMSTF) and the European Society of Gastrointestinal Endoscopy (ESGE) guidelines [52,53].

## Methods

### Colonoscopy Database

We included all adult medical patients who had undergone colonoscopy at the Queen Mary Hospital of Hong Kong, a major regional hospital, between February 1, 2016, and May 7, 2020 (Table 1). Patients who were older than 80 years, had colorectal cancer, inflammatory bowel disease, a history of colectomy, an incomplete colonoscopy, poor bowel preparation with inadequate bowel preparation according to the Aronchick scale, a prior colonoscopy, a family history of colorectal cancer or hereditary syndrome associated with increased risk, or serrated polyposis syndrome were excluded. Patient’s baseline demographic and endoscopic findings including the number, size, and final pathology of each detected polyp were retrieved. All resected polyps were histologically reported according to the World Health Organization criteria as standard hospital practices. Advanced adenomas were defined as adenomas ≥10 mm in diameter, with villous histology in at least 25% of the adenoma, or high-grade dysplasia or carcinoma. The advanced serrated polyp was defined as a sessile serrated polyp with dysplasia, or ≥10 mm in diameter, or a traditional serrated adenoma.

**Table 1:** Baseline characteristics of all patients (N=3735).

Patient’s demographic and endoscopic findings		Values
**Demographic**		
	Age (years), median (IQR)	62.6 (53-69)
	Male sex, n (%)	1971 (52.7)
	Smoker, n (%)	299 (8)
	Drinker, n (%)	627 (16.7)
**Comorbid conditions, n (%)**		
	Ischemic heart disease	722 (19.3)
	Diabetes mellitus	711 (19)
**Indication for colonoscopy**		
	Screening, n (%)	805 (21.5)
	Symptomatic, n (%)	2930 (78.4)
	Bowel preparation score (Aronchick scale), median (IQR)	2 (2-3)
	Withdrawal time in minutes, mean (95% CI)	12.2 (12.0-12.4)
**Endoscopic findings**		
	Polyp detection rate (%), rate (95% CI)	50.6 (49.9-51.3)
	Number of polyps detected per patient, mean (95% CI)	1.20 (1.17-1.23)
	Adenoma detection rate (%), rate (95% CI)	38 (37.1-38.7)
	Number of adenomas detected per patient, mean (95% CI)	0.81 (0.79-0.84)
	Advanced adenoma^a^ detection rate (%), rate (95% CI)	19.9 (19.3-20.5)
	Number of advanced adenomas detected per patient, mean (95% CI)	0.37 (0.35-0.38)
	Serrated polyp detection rate (%), rate (95% CI)	16.6 (16-17.2)
	Number of serrated polyps detected per patient, mean (95% CI)	0.25 (0.24-0.26)
	Advanced serrated polyp^b^ detection rate (%), rate (95% CI)	2 (1.7-2.2)
	Number of advanced serrated polyps detected per patient, mean (95% CI)	0.02 (0.01-0.03)

^a^Advanced adenoma was defined as an adenoma of 10 mm, with the presence of tubulovillous or villous histology or high-grade dysplasia.

^b^Advanced serrated polyp was defined as serrated polyps 10 mm, sessile serrated polyps with dysplasia, and traditional serrated adenomas.

### Predicted Changes in Surveillance Interval

We first determined the baseline recommended surveillance interval for each patient based on their original colonoscopy and histological findings, according to the USMSTF or the ESGE guideline [52,53]. This was followed by a simulation of the projected increase in the detection of colonic lesions with the application of CADe as reported in a recent meta-analysis of randomized controlled trials, which was 0.42 (95% CI 0.33-0.50) for an additional increase in the mean number of polyps and serrated polyps, and 0.18 (95% CI 0.13-0.22) for an additional increase in the mean number of adenomas detected per colonoscopy. The projected increase in the polyp or serrated polyp detection rate was 15.1% (95% CI 11.1-17.8), and the adenoma detection rate was 9.7% (95% CI 7.0-12.9) [8] as calculated by the raw data from the same meta-analysis. There was no increase in the detection of advanced adenomas and advanced serrated polyps (Table 2).

We assumed the improvement in the detection of polyps, adenomas, and advanced adenomas by CADe followed the Poisson distribution. The performance of CADe was randomly generated according to this distribution by bootstrapping 200 steps, which was used to simulate the effect of CADe on each patient with the predicted number of polyps and the size and histology of each polyp. For each bootstrapping step, the distribution of patients in each risk category was generated based on the projected endoscopic findings after the application of CADe under the current USMSTF or ESGE guideline. The mean proportion of patients in each risk category for all bootstrapping steps was used to generate the final result. The corresponding changes in the recommended colonoscopy surveillance interval if CADe was applied were then compared with the baseline findings.

**Table 2:** Assumptions of computer-aided detection performance on our simulated population.

Assumptions	CADe^a^ performance
Mean increase in polyp detection rate (%), rate (95% CI)	15.1 (11.1-17.8)
Mean increase in adenoma detection rate (%), rate (95% CI)	9.7 (7.0-12.9)
Mean increase in serrated polyp detection rate (%), rate (95% CI)	15.1 (11.1-17.8)
Mean increase in polyp detection per colonoscopy, mean (95% CI)	0.42 (0.33-0.50)
Mean increase in adenoma detection per colonoscopy, mean (95% CI)	0.18 (0.13-0.22)
Mean increase in serrated polyp detection per colonoscopy, mean (95% CI)	0.42 (0.33-0.50)
Detection for advanced adenomas	No increase
Detection of advanced serrated polyps	No increase

^a^CADe: computer-aided detection.

### Outcome Measures

The primary outcome of interest was the projected changes in surveillance intervals with the increase in colonic lesion detection after the application of CADe. Specifically, we determined the changes in proportions of patients in each recommended surveillance interval; the number of patients who would have to shorten the surveillance interval; the number of patients with changes in surveillance due to an increase in adenoma detection; and the number of patients with changes in surveillance due to an increase in sessile serrated lesion detection after the application of CADe. Changes in surveillance intervals according to the USMSTF or the ESGE recommendations were analyzed separately. The McNemar-Bowker Test of Symmetry was used to compare the changes before and after the CADe application.

### Ethical Considerations

The study protocol was approved by the institutional review board of the University of Hong Kong and the West Cluster of the Hong Kong Hospital Authority (reference number UW 20-279).

## Results

### Baseline Findings Without the Use of CADe

A total of 3735 patients with complete colonoscopy findings were included. Their baseline characteristics were summarized in Table 1. The median age was 62.6 (IQR 53.0-69.0) years, with 52.7% (n=1971) male participants. Among them, 8% (n=299) were chronic smokers, 16.8% (n=627) were chronic drinkers, 19.3% (n=722) had ischemic heart disease, and 19% (n=711) had diabetes mellitus. A majority (78.4%, n=2930) of patients underwent colonoscopy for symptoms, and 21.5% (n=805) underwent screening colonoscopy. The median Aronchick score of bowel cleanliness was 2 (IQR 2-3), and the mean withdrawal time was 12.2 (95% CI 12.0-12.4) minutes.

For the whole cohort, the polyp detection rate was 50.6% (95% CI 49.9-51.3), and the mean number of polyps detected per patient was 1.20 (95% CI 1.17-1.23). The adenoma detection rate was 38% (95% CI 37.1-38.7), and the mean number of adenomas detected per patient was 0.81 (95% CI 0.79-0.84). The serrated polyp detection rate was 16.6% (95% CI 16.0-17.2), and the number of serrated polyps detected per patient was 0.25 (95% CI 0.24-0.26). The advanced adenoma detection rate was 19.9% (95% CI 19.3-20.5), whereas the advanced serrated polyp detection rate was 2% (95% CI 1.7-2.2). The projected increase in the detection of various lesions after CADe was shown in Table 3.

**Table 3:** Projected improvement in polyp and adenoma detection after application of CADe^a^.

Endoscopic findings	Baseline	After CADe
Polyp detection rate (%), rate (95% CI)	50.6 (49.9-51.3)	64.6 (63.0-66.3)
Number of polyps detected per patient, mean (95% CI)	1.20 (1.17-1.23)	2.66 (2.47-2.88)
Adenoma detection rate (%), rate (95% CI)	38 (37.1-38.7)	47.7 (45.9-50.0)
Number of adenomas detected per patient, mean (95% CI)	0.81 (0.79-0.84)	1.69 (1.56-1.85)
Advanced adenoma detection rate (%), rate (95% CI)	19.9 (19.3-20.5)	19.9 (19.3-20.5)
Number of advanced adenomas detected per patient, mean (95% CI)	0.37 (0.35-0.38)	0.37 (0.35-0.38)
Serrated polyp detection rate (%), rate (95% CI)	16.6 (16-17.2)	31.6 (28.4-34.5)
Number of serrated polyps detected per patient, mean (95% CI)	0.25 (0.24-0.26)	0.49 (0.45-0.52)
Advanced serrated polyp detection rate (%), rate (95% CI)	2 (1.7-2.2)	2 (1.7-2.2)
Number of advanced serrated polyps detected per patient, mean (95% CI)	0.02 (0.01-0.03)	0.02 (0.02-0.03)

^a^CADe: computer-aided detection.

### Effect of CADe on Surveillance Interval According to the Current USMSTF Guideline

**Figure 1:**
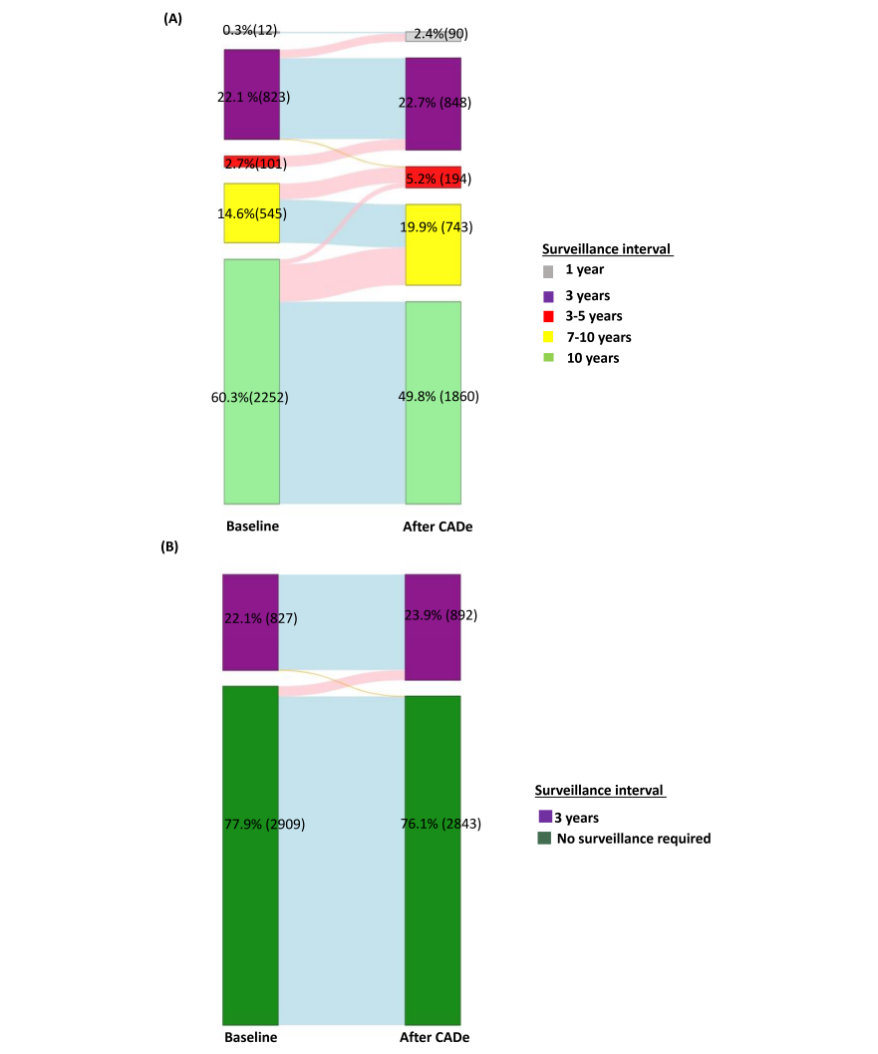
Sankey diagram showing the projected changes in the proportion of patients with different surveillance intervals after CADe application according to (A) the US Multi-Society Task Force on Colorectal Cancer (USMSTF) and (B) the European Society of Gastrointestinal Endoscopy (ESGE) recommendations. CADe: computer-aided detection.

Following the current USMSTF recommendation, 19.1% (95% CI 17.3%-21.2%; n=714, 95% CI 646-791) of all patients would have shorter surveillance intervals after CADe application as compared to baseline findings (Figure 1A). The overall proportion of patients who would require 1-year surveillance increased from 0.3% (12/3735) to 2.4% (88/3735; 95% CI 2%-3.4%), and the proportion of patients who would require 3-5 years of surveillance increased from 2.7% (101/3735) to 5.1% (192/3735; 95% CI 4.9%-5.6%). Accordingly, the proportion of patients who would require 7-10 years of surveillance increased from 14.5% (545/3735) to 19.9% (743/3735; 95% CI 18%-22.1%), and the proportion of patients who would require 10 years of surveillance decreased from 60.3% (2251/3735) to 49.8% (1861/3735; 95% CI 47.8%-51.7%). In the first 3 years, the projected increase in the number of surveillance colonoscopies was from 299 to 312 (95% CI 291-335), or a 4% increase per year.

**Figure 2:**
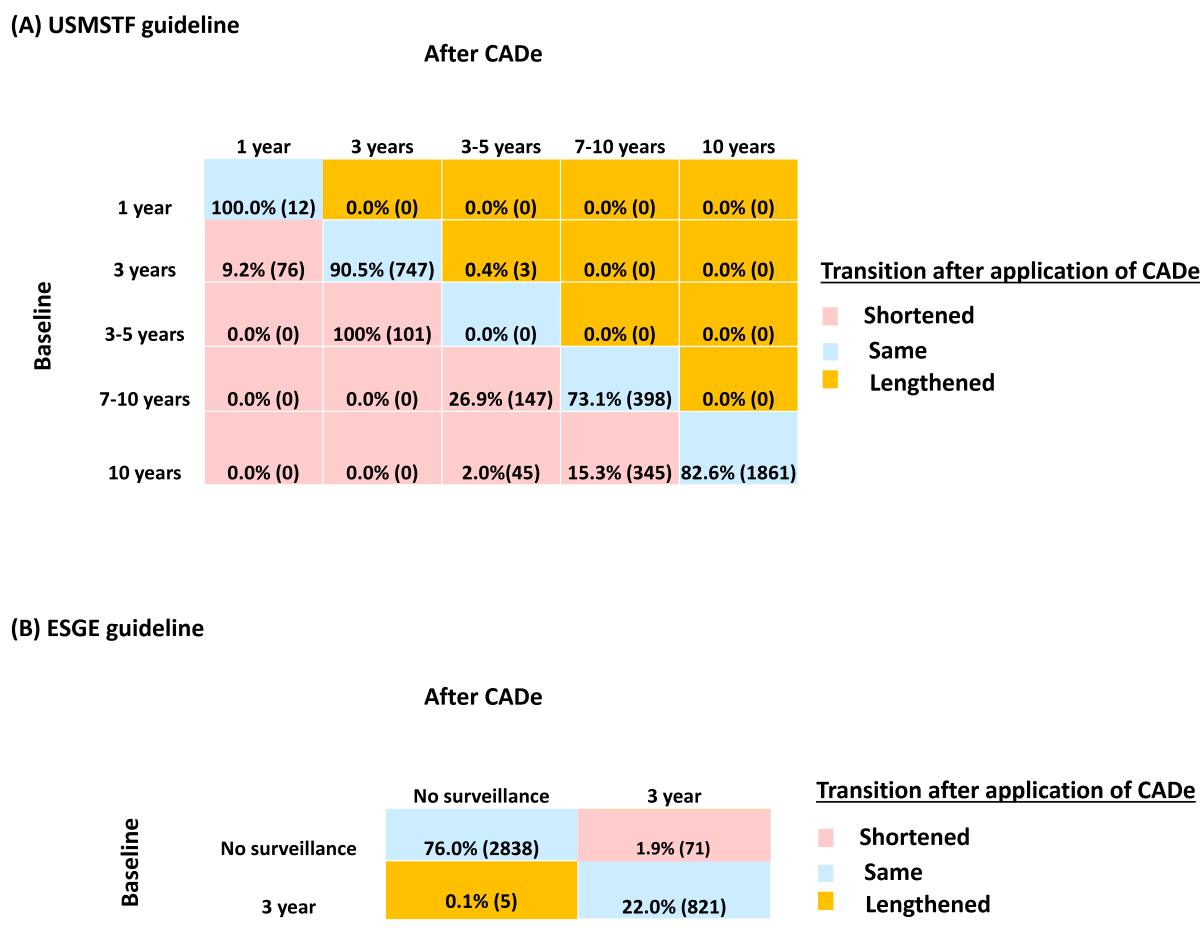
Percentage of patients undergoing conventional colonoscopy who required changes in different surveillance intervals after the CADe application according to (A) the USMSTF and (B) the ESGE guidelines. CADe: computer-aided detection; ESGE: European Society of Gastrointestinal Endoscopy; USMSTF: US Multi-Society Task Force on Colorectal Cancer.

The shifts in screening intervals after CADe application according to baseline intervals were shown in Figure 2A. The most notable change was observed in patients who were originally scheduled to have surveillance for 3-5 years, in which all (n=101 or 2.7% of the total) patients were shortened to 3 years after CADe. Among them, 74% (75/101) and 26% (26/101) were contributed by the increase in the detection of adenoma and serrated lesions, respectively. The second most notable group was patients who were initially recommended to have 7-10 years of surveillance; 26.9% (n=147 or 3.9% of the total) of these patients would be shortened to 3-5 years of surveillance. The changes were attributed to an increase in adenoma detection in 98.6% (145/147) of cases, with the rest due to an increase in serrated lesions detection. In contrast, only 2% (n=45 or 1.2% of the total) of patients who were originally assigned to 10 years of surveillance would be shortened to 3-5 years of surveillance, and 15.3% (n=345 or 9.2% of the total) of these patients would be shortened to 5-7 years of surveillance.

In patients with advanced adenomatous or serrated lesions at baseline, 7.5% (n=60) would have shorter surveillance intervals after CADe, which was significantly lower than those without baseline advanced lesions (22.3%, n=655; *P*<.001).

### Effect of CADe on Surveillance Interval According to the Current ESGE Guideline

Based on the current ESGE recommendation, a total of 23.9% (95% CI 23.2%-24.5%; n=892, 95 CI 868-915) of patients would need 3 years of surveillance if CADe was applied (Figure 1B), as compared to 22.1% (821/3735) originally. Specifically, 71 (95% CI 62-80) or 1.9% of the total (95% CI 1.6%-2.1%) patients would change from no surveillance to 3-year surveillance after CADe (Figure 2B). Among these patients, 94.3% (67/71) and 5.6% (4/71) of changes were due to an increase in adenoma and serrated lesion detection, respectively.

When compared to the changes according to the USMSTF guideline, the proportion of patients requiring a shortening of the surveillance interval was significantly lower under the ESGE guideline (19.1%, 714/3735 vs 1.9% 71/3735; *P*<.001). In the first 3 years, the projected increase in the number of surveillance colonoscopies would be from 275 to 297 (95% CI 289-305), or 8% per year.

According to the ESGE guideline, none of the patients with advanced adenomatous or serrated lesions at baseline would have shorter surveillance intervals after CADe, which was significantly lower than those without baseline advanced lesions (2.4%, n=72; *P*<.001).

## Discussion

### Principal Findings

In this simulation study based on our large cohort of patients who had undergone colonoscopy, we showed that 19.1% (714/3735) of patients would have shorter surveillance intervals according to the USMSTF guideline if CADe were routinely applied. Accordingly, more patients would require 1 year (0.3% to 2.3%) and 3-5 years (2.7% to 5.2%) of surveillance. Among patients who were originally scheduled to have 3-5 years of surveillance, all of them were shortened to 3 years after CADe. In contrast, only 1.9% (71/3735) of patients would have their surveillance intervals changed from no surveillance to 3-year surveillance according to the ESGE guideline.

Due to the differences in the recommendation of surveillance intervals after index colonoscopy between the 2 guidelines, it appears that the CADe application would affect the surveillance intervals following the current USMSTF recommendation. In the USMSTF recommendation, patients are stratified according to the size, number, and histology of the polyps into 6 different surveillance intervals [52]. In contrast, the latest ESGE recommendation had only 2 strata, in which surveillance was not recommended in patients with complete removal of less than 5 small (<10 mm) adenomas with low-grade dysplasia, regardless of the presence of villous components, or any small serrated polyp without dysplasia [53]. Some of these patients, however, would be recommended to have 3-5 or 5-10 years of surveillance under the current US guideline.

Although CADe could also increase sessile serrated lesion detection, we showed that the shortening of surveillance intervals was largely attributed to the increase in adenoma rather than serrated lesion detection. This is most likely related to the relatively low background prevalence (16.6%) of serrated lesions in our cohort, as well as the dominance or co-occurrence of adenomatous lesions with serrated lesions in general (Table 1).

The increase in colonic lesion detection by the CADe resulting in the shortening of the surveillance interval will inevitably increase the demand for subsequent surveillance colonoscopies. In the initial 3 years, it is anticipated that there will be a 4%-8% increase in the number of surveillance colonoscopies in our cohort. While the demand for colonoscopies continues to increase in most countries due to the rising number of screening and surveillance colonoscopies, the associated burden on the health care system cannot be overlooked. As in our center, the waiting time for routine colonoscopies has progressively lengthened over the past few years, even before the wide adoption of CADe, despite an overall increase in colonoscopy throughput. However, most of the incremental polyps or adenomas detected by CADe are small in size [6], and it remains to be determined whether the application of CADe and the associated increase in surveillance frequency would ultimately translate into a further reduction in colorectal cancer incidence and mortality in the real world. To this end, a recent modeling study suggested that the application of CADe could further reduce the colorectal cancer incidence and mortality of screening colonoscopy with an incremental gain of 4.8% and 3.6%, respectively [47]. Intuitively, the detection and subsequent removal of more adenomas by CADe could already lower the subsequent cancer risk, which may make the subsequent surveillance colonoscopy less important than patients undergoing an index colonoscopy. There may be a need to reconsider the surveillance intervals after colonoscopy with CADe in the future.

### Limitations

This study has limitations. First, while the performance of different CADe models could vary with different populations and the performance of endoscopists, we chose the summary results reported in a recent meta-analysis based on published randomized controlled studies [8]. However, bootstrapping the performance of the CADe system could account for the possible variable performance of different CADe systems. Second, only 21% (805/3735) of our patients underwent colonoscopy for screening purposes, which could account for the relatively high advanced adenoma detection rate (19.9%). However, current surveillance intervals are based on endoscopic findings, irrespective of indications for colonoscopy. Furthermore, as shown in our study, use of CADe is more likely to shorten surveillance intervals in those with no advanced lesions at baseline than in those with advanced lesions. Hence, our results could still possibly underestimate the impacts of CADe application on surveillance intervals if applied to the screening cohort. Third, this study did not address the application of CADe to potential changes in subsequent risk categories for individual patients. Recent multicenter randomized controlled trials showed that CADe could effectively reduce both the polyp and adenoma miss rates by almost 50% [43,54], which could potentially reduce the occurrence of metachronous advanced lesions on follow-up endoscopy. As one of the reasons for the finding of metachronous advanced lesions on surveillance colonoscopy was the presence of missed lesions [55-59], the application of CADe could help to reduce the number of advanced lesions detected on future colonoscopies, and possibly postcolonoscopy colorectal cancer. Fourth, this study did not address the cost-effectiveness of the use of CADe. In a recent modeling study [47], it was suggested that the implementation of CADe during screening colonoscopies could result in a yearly saving of US $290 million at the US population level. As of yet, the additional benefits of these extra surveillance colonoscopies incurred by the CADe remain to be addressed in real clinical practices, including our local practices where the CADe is not routinely applied.

### Conclusions

Our simulation study showed that the wide adoption of CADe during colonoscopy and the associated increase in colonic lesion detection could lead to a shortening of subsequent colonoscopy surveillance intervals. In particular, 19.1% (714/3735) of patients would require a shortening of the surveillance interval if they follow the USMSTF guideline, but only 1.9% (71/3735) of patients if they follow the current ESGE guideline. With mounting evidence that favors the enhanced diagnostic role of CADe and the further reduction in risk of colorectal cancer, future studies may be necessary to reconsider the appropriate surveillance intervals after the application of CADe.

## Abbreviations

CADe: computer-aided detection
ESGE: European Society of Gastrointestinal Endoscopy
USMSTF: US Multi-Society Task Force on Colorectal Cancer

## Data Availability

The data set used in this study is not publicly available. However, some data from this study can be provided upon reasonable request to the corresponding author.
